# Sericin as treatment of obesity: morphophysiological effects in obese mice fed with high-fat diet

**DOI:** 10.31744/einstein_journal/2020AO4876

**Published:** 2019-09-20

**Authors:** Regina Inês Kunz, Angélica Novi Capelassi, Ana Cláudia Paiva Alegre-Maller, Maria Lúcia Bonfleur, Lucinéia de Fátima Chasko Ribeiro, Rose Meire Costa, Maria Raquel Marçal Natali

**Affiliations:** 1 Universidade Federal da Fronteira Sul Passo FundoRS Brazil Universidade Federal da Fronteira Sul, Passo Fundo, RS, Brazil.; 2 Universidade Estadual do Oeste do Paraná CascavelPR Brazil Universidade Estadual do Oeste do Paraná, Cascavel, PR, Brazil.; 3 Universidade Estadual do Maringá MaringáPR Brazil Universidade Estadual do Maringá, Maringá, PR, Brazil.

**Keywords:** Bombyx mori, Obesity/drug therapy, Sericins/therapeutic use, Liver, Intestine, small, Jejunum, Diet, high-fat, Mice

## Abstract

**Objective:**

To investigate the effects of sericin extracted from silkworm *Bombyx mori* cocoon on morphophysiological parameters in mice with obesity induced by high-fat diet.

**Methods:**

Male C57Bl6 mice aged 9 weeks were allocated to one of two groups - Control and Obese, and fed a standard or high-fat diet for 10 weeks, respectively. Mice were then further subdivided into four groups with seven mice each, as follows: Control, Control-Sericin, Obese, and Obese-Sericin. The standard or high fat diet was given for 4 more weeks; sericin (1,000mg/kg body weight) was given orally to mice in the Control-Sericin and Obese-Sericin Groups during this period. Weight gain, food intake, fecal weight, fecal lipid content, gut motility and glucose tolerance were monitored. At the end of experimental period, plasma was collected for biochemical analysis. Samples of white adipose tissue, liver and jejunum were collected and processed for light microscopy analysis; liver fragments were used for lipid content determination.

**Results:**

Obese mice experienced significantly greater weight gain and fat accumulation and had higher total cholesterol and glucose levels compared to controls. Retroperitoneal and periepididymal adipocyte hypertrophy, development of hepatic steatosis, increased cholesterol and triglyceride levels and morphometric changes in the jejunal wall were observed.

**Conclusion:**

Physiological changes induced by obesity were not fully reverted by sericin; however, sericin treatment restored jejunal morphometry and increased lipid excretion in feces in obese mice, suggesting potential anti-obesity effects.

## INTRODUCTION

Obesity is a global epidemic characterized by excess body fat and cardiometabolic complications resulting from excess intake of calories, sugar, salt and fat combined with increasingly passive labor and entertainment activities.^1^

The small intestine is responsible for effective absorption and processing of ingested nutrients.^2 , 3^ It is also the first organ to be exposed to food energy^4^ and is capable of functional and morphological adaptation in response to ingested food quantity and quality.^5^ Obesity, particularly when induced by high-fat and/or high-calorie diets, has been shown to affect intestinal wall components.^3 , 5 , 6^

Along with fat tissue changes and occasional small intestine adaptations, obesity *per se* is a risk factor for other conditions, including non-alcoholic fatty liver disease (NAFLD) - the most common chronic disease affecting the liver.^7 , 8^

Lifestyle changes, such as improved dietary habits and physical activity practice, are the major therapeutic alternatives for obesity and NAFLD.^7 , 9^ However, individual responses to these strategies may not suffice.^10^

In these settings, potential natural anti-obesity agents with less adverse effects, such as the biopolymer sericin produced by the silkworm *Bombyx mori* ( *Lepidoptera, Bombycidae* ), are being increasingly sought after.^7 , 11^ Sericin is a natural, highly hydrophilic globular protein, with molecular weight ranging from 20 to 400kDa, which, together with fibroin, forms the silk thread.^12^ Physicochemical characteristics of the sericin molecule have been associated with several properties with potential biologic applicability.^12 - 14^

Combined sericin and high-fat diet intake has been associated with improved glucose tolerance and hypolipidemic effects,^15^ increased high density lipoprotein (HDL) levels and regulation of cytokine production by fat tissues, leading to reduced leptin, resistin and tumor necrosis factor alpha (TNF-α) levels, and increased plasma adiponectin levels.^16^ Inhibition of cholesterol absorption by intestinal cells^17^ and antioxidant properties^18^ have also been reported. Sericin is thought to be a highly promising obesity prevention strategy; however, its therapeutic effects on established obesity and related comorbidities remain to be determined.

## OBJECTIVE

To investigate the effects of sericin on plasmatic parameters and adipose, hepatic and intestinal tissue morphology in mice with fat-rich diet-induced obesity.

## METHODS

This study was conducted at *Universidade Estadual do Oeste do Paraná* from July 2014 to December 2016.

### Sericin extraction and amino acid content determination

Sericin was extracted from *Bombyx mori* cocoons obtained from the silk farming company BRATAC S.A (Londrina, PR, Brazil). Cocoons were cut into pieces measuring approximately 1cm^2^ and sericin extraction (6g of cocoon per 100mL of distilled water) performed under high temperature and pressure conditions (120°C and 1kgf/cm^2^ respectively) in autoclave (CS 30, Prismatec, Itu, SP, Brazil) for 60 minutes. No chemical additives were used. Fibroin was separated using an 18-mesh sieve; sericin solution was frozen (-20°C) and sericin powder obtained by freeze-drying (LT 1000, Terroni Equipamentos Ltda., São Carlos, SP, Brazil).

Sericin soluble amino acid content was determined by ultra-performance liquid chromatography (UPLC). UPLC was run at *Laboratório de Bioquímica e Biofísica do Instituto Butantan* (São Paulo, SP, Brazil) using a C18 reverse phase analytical column coupled to a detector with wavelength detection set at 214nm.

### Animals and diet

Procedures in this study were approved by the institutional Animal Ethics Committee (April 11^th^, 2014).

Male C57Bl/6 mice aged 9 weeks on average and weighing 26.9±2.2g were used. Mice were housed in a controlled environment (23 to 25°C and 12-hour light-dark cycle) and allocated to one of two groups following a one-week acclimation period, as follows: Control (n=14) – standard rodent diet (Algomix^®^, Algomix Agroindustrial Ltda., Ouro Verde do Oeste, PR, Brazil) and water; Obese Group (n=14) – fat-rich diet and water. Diets were fed for 10 weeks and mice then further divided into four experimental groups according to sericin treatment (Control, Control-Sericin, Obese and Obese-Sericin) and comprising seven mice each. Water, standard rodent diet and the fat-rich diet were fed *ab libitum* throughout the experimental period.

Fat-rich diet ingredients were outsourced from Prag Soluções (Jaú, SP, Brazil) and consisted of 30.07% corn starch, 14% casein, 12% sucrose, 4% soil bean oil, 5% microcrystalline cellulose, 3.5% AIN-93M mineral mix, 1.0% AIN-93M vitamin mix, 0.18% L-cystine, 0.25% choline bitartrate, and 30% lard. Feed containing 32.21% of carbohydrate, 10.6% of protein and 57.2% of lipid was given in pellet form.

Sericin treatment (Control-Sericin and Obese-Sericin groups) was introduced following a 10-week period of consumption of the standard or fat-rich diet. Sericin (1,000mg/kg of body weight) was fed daily at the same hour, for 4 consecutive weeks. Sericin was fed by gavage and the daily dose diluted in 300μL of 0.9% saline. Control and Obese Group mice were fed 300μL of pure 0.9% saline by gavage.

### Body weight measurement, feed intake control and fecal sampling

Individual body weight was monitored weekly. In the last week of the experimental period, mice were placed in individual metabolic cages for three consecutive 12-hour periods for feed intake determination. Mean feed intake was then calculated and fecal samples collected, weighed, frozen and used for total lipid content determination.^19^

### Bowel transit time test

Mice were fed 300µL of marker consisting of 3g of carmine in 50mL of 0.5% ethylcellulose. Marker was fed by gavage four days prior to euthanasia. Bowel transit time determination was based on time from gavage to first stained (pink-red) fecal pellet output.^20^

### Oral glucose tolerance test

Mice were submitted to oral glucose tolerance test (oGTT) two days prior to euthanasia. After 8 hours of fasting, mice were weighed and blood samples collected form the tail for fasting glucose level determination (time point 0) using a glucometer (Accu-Chek Active^®^, Roche Diagnóstica Brasil Ltda., SP, Brazil). Mice were then fed glucose (2g/kg of body weight) by gavage and glucose levels measured within 15, 30, 60 and 120 minutes of administration. The area under the curve (AUC) was calculated per mouse using GraphPad Prism 6.0 software.

### Plasma level determinations

Mice were submitted to a 12-hour fasting period, anesthetized with ketamine hydrochloride and xylazine (100mg/kg and 10mg/kg respectively) via the intraperitoneal route, and euthanized by exsanguination via the orbital plexus using a heparinized capillary. Blood samples were spun (10,000rpm/10 minutes at 4ºC) and the plasma fraction extracted. Triglyceride and total cholesterol levels and alanine and aspartate aminotransferase (ALT and AST) levels were measured using commercial kits (Laborclin^®^, Bioliquid, PR, Brazil and Analisa^®^, Gold Analisa Diagnóstica Ltda., Belo Horizonte, MG, Brazil, respectively) according to manufacturer’s instructions. Glucose levels were measured using a glucometer.

### Obesity assessment

At the end of the 14-week experimental period and at the age of 160 days, on average, mice were weighed and the retroperitoneal and periepidydimal fat pads removed and weighed for obesity determination.

### Morphological studies

Retroperitoneal and periepidydimal fat samples were fixed using 4% paraformaldehyde in phosphate buffer saline (PBS) for 24 hours, dehydrated in increasing concentrations of alcohol, diaphanized in xylol and embedded in paraffin. Tissue sections (5µm-thick) were stained with hematoxylin and eosin (HE). An area corresponding to 100 randomly distributed adipocytes was measured per section (10 points each) per fat type per animal using Image Pro Plus^®^ 6.0 software (Media Cybernetics, USA).

The liver was removed from the abdominal cavity and weighed for hepatic mass determination. Samples from the right lobe were collected, fixed using 4% paraformaldehyde in PBS for 24 hours, then processed for paraffin embedding. Tissue sections (5µm thick) were stained with hematoxylin and eosin (HE) or Masson’s trichrome (overall morphology and connective tissue analysis, respectively). Steatosis was graded zero to 3 Kleiner et al.,^21^ in a double-blind fashion, as follows: 0, up to 5%; 1, 5 to 33%; 2, 33 to 66% and 3, over 66%.

The small intestine was stretched out and the total length measured. Jejunal samples were opened at the mesenteric border, fixed using 4% paraformaldehyde in PBS for 24 hours, then submitted to routine histological processing for paraffin embedding. Semi-serial 6µm-thick sections were stained with HE for morphological and morphometric analysis, or with the periodic acid-Schiff histochemical method for goblet cell count.

Morphometric analysis of jejunal samples was conducted using previously calibrated Image Pro Plus^®^ 6.0. Intestinal wall thickness and villus height were measured under 200x magnification; measurements were made at 10 randomly selected points, 30µm apart per section, totaling up 50 measurements per animal. Tunica muscularis thickness, crypt depth and villus width were measured under 400x magnification (50 measurements per animal per analysis).

Goblet cells were counted in intestinal villi. The number of goblet cells and the total number of cells on the one side of the villus were counted up to approximately 1,500 cells per animal. Therefore, the percentage of goblet cells corresponded to the total goblet cell count adjusted for the total cell count.

### Hepatic lipid profile determination

Liver fragments weighing approximately 500mg were collected from each mouse and immediately frozen. Hepatic lipids were then extracted^19^ and cholesterol and triglyceride levels determined using the same kits employed for plasma level determinations.

### Statistical analysis

Results were expressed as mean±standard error of the mean and analyzed using GraphPad Prism 6.0 software. Data normality was confirmed using the Shapiro-Wilk test; two-way Analysis of Variance (ANOVA) followed by the Tukey’s post-hoc test were then performed. The level of significance was set at p<0.05.

## RESULTS

### Sericin amino acid content

Sericin chromatographic profile (UPLC) revealed 17 amino acids, with higher percentages of serine, glycine and aspartic acid ( Table 1 ).

**Table 1:** Serin amino acid content

Amino acid	Molar percentage
Aspartic acid	14.5
Glutamic acid	4.85
Serine	30.37
Glycine	27.16
Hystidine	2.31
Arginine	2.55
Threonine	1.97
Alanine	0.73
Proline	2.61
Tyrosine	2.89
Valine	1.47
Methionine	0.19
Cysteine	0.84
Isoleucine	0.81
Leucine	2.54
Phenylalanine	0.17
Lysine	4.04

### Body weight changes

**Figure 1:**
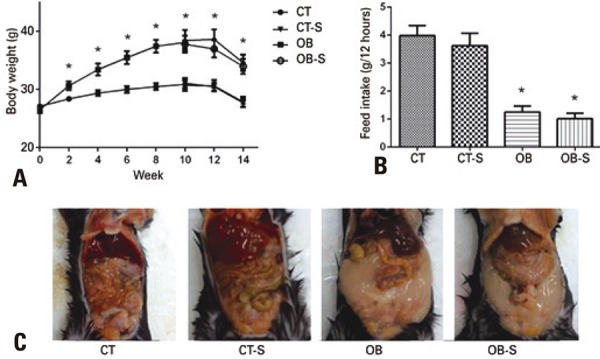
Body weight changes. Graph displaying body weight changes (A) and feed intake (B), and photographic images of the inner aspect of the abdominal cavity of C57Bl6 mice (C)

Mice fed the fat-rich diet experienced significant body weight gain from the second week to the end of the 14-week experimental period compared to mice fed the standard diet ( Figure 1A ). The fat-rich diet significantly reduced feed intake compared to the standard diet ( Figure 1B ), in spite of increased adiposity, as shown in mice abdominal cavity images ( Figure 1C ).

Results expressed as mean±standard error of the mean (n=7/group). * p<0.01 compared to CT and CT-S; two-way Analysis of Variance and Tukey’s post-hoc test.

CT: control; CT-S: control-sericin; OB: obese; OB-S: obese-sericin.

### Bowel transit time, fecal weight and fecal lipid content

**Figure 2:**
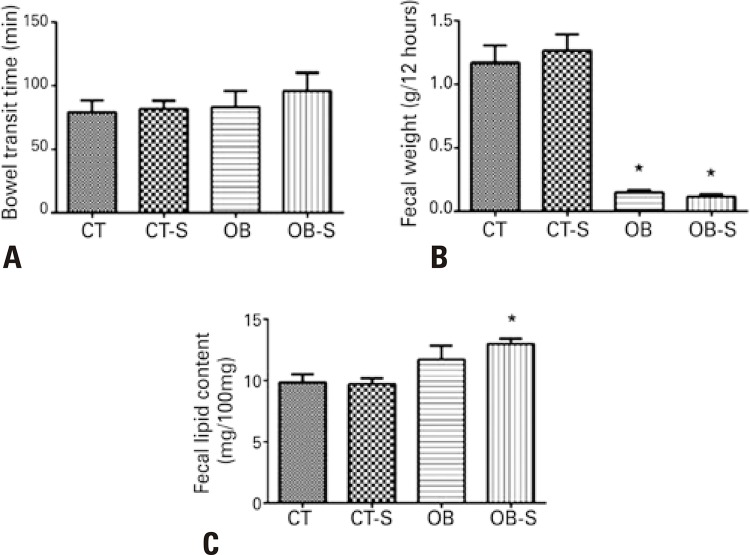
Analysis of bowel transit time and fecal parameters. Bowel transit time (A), fecal weight (B), fecal lipid content (C)

Diet-induced obesity and sericin treatment did not interfere with mice bowel transit time ( Figure 2A ); however, the fecal volume was significantly smaller in mice fed the fat-rich diet. This was directly related to lower feed intake in these groups, regardless of sericin treatment ( Figure 2B ). Fecal lipid content was also higher in the Obese-Sericin Group compared to mice fed the standard diet ( Figure 2C ).

Results expressed as mean±standard error of the mean (n=7/group). * p<0.01 compared to CT and CT-S. Two-way Analysis of Variance and Tukey’s post-hoc test.

CT: control; CT-S: control-sericin; OB: obese; OB-S: obese-sericin.

### Oral glucose tolerance test

**Figure 3:**
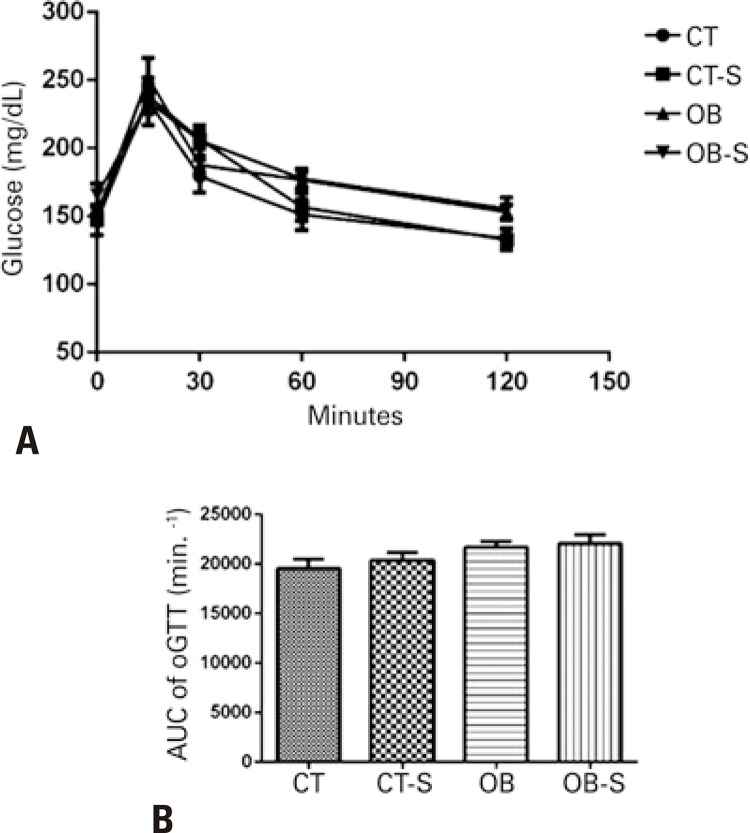
Results of the oral glucose tolerance test. Glucose values during the oral glucose tolerance test (A), area under curve of oral glucose tolerance test (B)

As shown in figure 3A , glucose peak during the oGTT occurred at 15 minutes in mice in all groups, with gradual drop over the course of the test and normalization within 120 minutes. The glucose AUC did not differ significantly between groups ( Figure 3B ).

Results expressed as mean±standard error of the mean (n=7/group). Two-way Analysis of Variance and Tukey’s post-hoc test.

CT: control; CT-S: control-sericin; OB: obese; OB-S: obese-sericin; oGTT: oral glucose tolerance test; AUC: area under the curve.

### Obesity model validation

Biometric parameters used to define obesity are shown in table 2 . Final body weight, carcass weight and retroperitoneal and periepidydimal fat deposits were impacted by fat-rich diet intake only, suggesting obesity development. Sericin treatment had no impact on these parameters.

**Table 2:** Biometric parameters

Biometric parameters	CT	CT-S	OB	OB-S
Final body weight, g	27.3±0.8	27.3±0.6	35.2±1.4*^†^	34.7±1.2*^†^
Carcass weight, g	19.9±0.7	20.3±0.6	25.6±1.2^*†^	25.1±1.0*^†^
Retroperitoneal fat, % body weight	0.26±0.04	0.37±0.08	2.06±0.29*^†^	1.98±0.21*^†^
Periepidydimal fat, % body weight	0.72±0.06	0.82±0.09	3.92±0.29*^†^	3.66±0.50*^†^

Results expressed as mean±standard error of the mean (n=7/group). Two-way Analysis of Variance and Tukey’s post-hoc test. * Statistically significant difference from CT; p<0.01; ^†^ Significant difference from CT-S; p<0.01.

CT: control; CT-S: control-sericin; OB: obese; OB-S: obese-sericin.

### Plasma parameters

Plasma glucose and total cholesterol levels were impacted by fat-rich diet intake, with statistically significant increase in obese compared to control mice; sericin treatment had no impact on these parameters. Plasma triglyceride and AST levels did not differ between groups, whereas ALT levels were higher in obese mice compared to mice fed the standard diet ( Table 3 ).

**Table 3:** Plasma parameters

Plasma parameters	CT	CT-S	OB	OB-S
Glucose, mg/dL	104.7±3.5	109.9±5.6	155.0±7.9*^†^	153.0±9.4*^†^
Cholesterol, mg/dL	81.7±5.5	83.8±3.2	115.7±8.2*^†^	127.0±7.2*^†^
Triglycerides, mg/dL	55.4±6.7	58.1±3.0	55.6±4.0	50.7±2.4
ALT, U/L	13.9±2.7	15.0±1.2	29.1±3.8*^†^	30.2±6.3*^†^
AST, U/L	36.0±4.0	34.4±4.6	41.6±6.8	37.9±4.9

Results expressed as mean±standard error of the mean (n=7/group). Two-way Analysis of Variance and Tukey’s post-hoc test. * Statiscally significant difference from CT; p<0.01; ^†^ Significant difference from CT-S; p<0.01.

CT: control; CT-S: control-sericin; OB: obese; OB-S: obese-sericin; ALT: alanine aminotransferase; AST: aspartate aminotransferase.

### Fat tissue morphology

**Figure 4:**
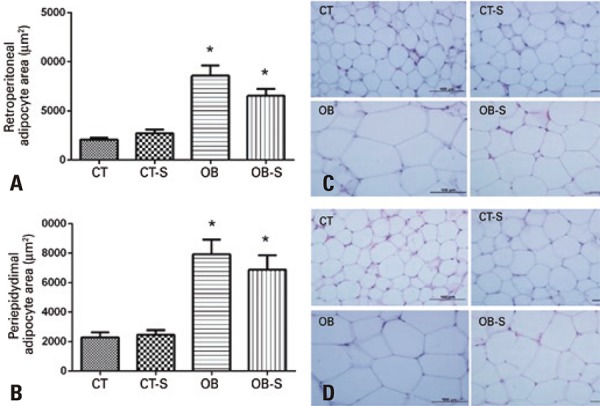
Fat tissue morphometry and morphology. Retroperitoneal (A) and periepidydimal (B) adipocyte area; photomicrographs of mice retroperitoneal (C) and periepidydimal (D) fat tissues; hematoxylin and eosin stain

Retroperitoneal and periepidydimal adipocyte areas were significantly larger in obese mice compared to mice fed the standard diet ( Figures 4A and 4B ). Sericin treatment had no significant impact on this parameter, in spite of subtle adipocyte area reduction in the Obese-Sericin Group ( Figures 4C and 4D ).

Results expressed as mean±standard error of the mean (n=7/group). Two-way Analysis of Variance and Tukey’s post-hoc test. * p<0.01 compared to CT and CT-S.

CT: control; CT-S: control-sericin; OB: obese; OB-S: obese-sericin.

### Liver morphology and hepatic lipid analysis

**Figure 5:**
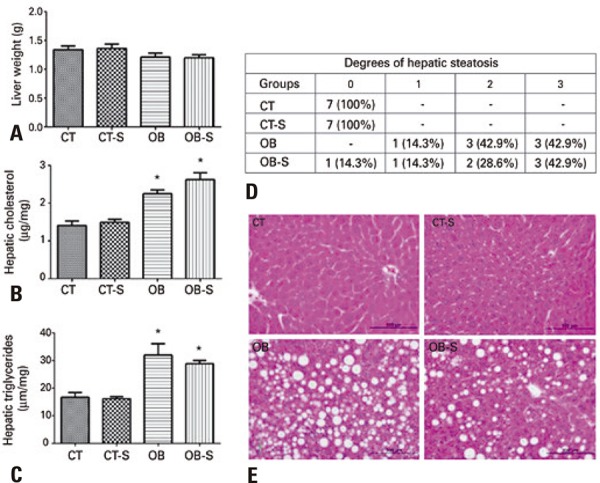
Liver morphology and hepatic lipid analysis. Liver weight (A), hepatic cholesterol (B) and triglyceride (C) analysis; hepatic steatosis classification (D), photomicrographs of mice liver; hematoxylin and eosin stain (E)

Liver weight did not differ significantly between groups ( Figure 5A ). Hepatic lipid profile analysis revealed higher hepatic cholesterol and triglyceride levels (p<0.0001) in obese compared to control mice ( Figures 5B and 5C ). Mice in the Control and Control-Sericin groups had no evidence of hepatic steatosis (grade zero), whereas varying degrees of steatosis were detected in the Obese and Obese-Sericin Groups ( Figure 5D ). Along with adipose vesicles ( Figure 5E ), balloon-shaped cytoplasm and occasional syncytium formation between adjacent hepatocytes were observed in some mice in the Obese and Obese-Sericin Groups.

Results expressed as mean±standard error of the mean (n=7/group). Two-way Analysis of Variance and Tukey’s post-hoc test. * p<0.01 compared to CT and CT-S.

CT: control; CT-S: control-sericin; OB: obese; OB-S: obese-sericin.

### Intestinal morphometry

Fat-rich diet intake led to changes in some intestinal morphometry parameters shown in table 4 . The small intestine was shorter in obese mice compared to mice fed the standard diet. Mice in the Obese Group had significantly longer villi (p<0.01; p<0.05) and reduced muscle layer thickness (p<0.01; p<0.05) compared to mice in the Control and Control-Sericin groups, respectively. Obese mice also had shallower crypts compared to controls (p<0.05). Changes promoted by fat-rich diet intake were reverted by sericin treatment in the Obese-Sericin Group. Intestinal wall thickness, villus width and goblet cell count did not differ between groups.

**Table 4:** Intestinal morphometry

Intestinal morphometry	CT	CT-S	OB	OB-S
Small intestine length, cm	39.7±0.9	39.9±0.6	33.1±0.9*^†^	33.8±0.9*^†^
Intestinal wall thickness, µm	496.4±17.9	504.7±24.0	558.7±30.6	513.0±17.7
Villus height, µm	306.0±19.9	327.9±13.6	390.2±18.8*^†^	356.4±8.4
Crypt depth, µm	96.2±3.0	90.2±4.0	78.5±5.6*	84.2±4.7
Villus width, µm	94.0±2.3	95.5±2.5	91.0±1.2	90.5±1.6
Tunica muscularis thickness, µm	51.1±3.6	50.1±3.87	37.2±1.6*^†^	41.1±1.9
Goblet cells, %	5.0±0.3	4.7±0.3	4.4±0.3	4.2±0.3

Results expressed as mean±standard error of the mean (n=7/group). Two-way Analysis of Variance and Tukey’s post-hoc test. * Statiscally significant difference from CT; ^†^ Significant difference from CT-S.

CT: control; CT-S: control-sericin; OB: obese; OB-S: obese-sericin.

## DISCUSSION

The C57Bl/6 mouse is an animal model of diet-induced obesity.^22^ The fat-rich diet in this study was able to effectively induce obesity, promoting body weight gain, fat deposit and increased plasma cholesterol and glucose levels despite lower feed intake. These findings are corroborated by the current literature,^23 - 26^ that is, saturated lipid-rich diets, such as the one used in this study, do in fact induce obesity.

Plasma triglyceride levels did not increase in obese mice despite higher lipid intake; similar findings have been reported in other studies with mice fed fat-rich diets^24 - 26^ and rats.^23^ Serum triglyceride levels may have remained unchanged due to redirection of these molecules to other tissues, particularly the adipose tissue, where they are stored.^27^ Adipocytes tend to respond to triglyceride accumulation with hypertrophy or hyperplasia.^28^ Increased retroperitoneal and periepidydimal fat pads and adipocyte area were observed in obese mice in this study. Adipocytes may also undergo homeostatic changes, such as adipose tissue hypoxia, macrophage infiltration, fibrosis and insulin resistance, which may lead to metabolic syndrome development.^29^

Remaining fat particles are cleared from the circulation by the liver,^27^ resulting in accumulation of significant amounts of lipids in obese individuals and ultimately in NAFLD.^8^ Higher liver triglyceride and cholesterol levels and fat deposits in the form or vesicles were detected in obese animals in this study. White vesicles in liver sections stained with HE confirmed the diagnosis of hepatic steatosis,^25^ a morphologic condition also reported in mice^24^ and rats.^18^ Biochemical analysis revealed increased serum ALT levels in obese mice in this trial. Recent studies based on similar animal models and diets differed with respect to the effects of obesity on hepatic transaminases. Sung et al.,^25^ failed to detect changes in transaminase levels, whereas Choi et al.,^30^ did not detect changes in ALT levels. Increased AST levels in obese mice fed fat-rich diets were the only change reported by Pang et al.^26^ Also, according to Abd El-Kader et al.,^8^ liver enzymes may be normal or minimally altered in NAFLD.

Apart from its vital role in nutrient digestion and absorption, the small intestine is capable of prompting adaptive changes in response to increased luminal lipid content;^4^ this may be related to metabolic syndrome progression in other organs.^31 , 32^ Reduced small intestinal length, increased villus height, shallower crypts and reduced jejunal tunica muscularis thickness were observed in mice fed the fat-rich diet in this trial. With the exception of tunica muscularis thickness, similar morphometric findings have been reported in Swiss mice fed a fat-rich diet for 8 weeks.^6^ Given feed intake was lower in obese mice, mucosal changes must be associated with food type^3^ rather than hyperphagia.^5^ Increased intestinal absorption area resulting from increased villus height^5^ may explain weight gain in obese mice. Liquid diets such as fat-rich diets may alter crypt morphology and reduce tunica muscularis thickness,^33^ as seen in this study, due to potential decrease in gut motility. Experimental evidence has shown that fat-rich diets may also induce neuron loss in the myenteric plexus,^34^ leading to reduced muscular thickness and gut motility. Reduced peristalsis is thought to be required for digestion and absorption of fat-rich diets; still, intestinal transit time and goblet cell count did not differ between groups in this trial, suggesting lack of functional changes. Goblet cells are responsible for mucus production for protection and lubrication of the intestinal epithelium.^3^

Sericin extraction in this study was performed with no chemical additives to produce a pure final product^18^ comprising 17 amino acids, with serine, glycine and aspartic acid accounting for more than 72% of sericin protein content. Amino acid content reflected previous descriptions^13 , 15 , 17^ and confirmed the hydrophilic nature of sericin. Lack of plasma or tissue morphology changes in mice in the Control-Sericin Group emphasizes the safety of sericin supplementation.^16 , 35^

Sericin treatment was not able to revert plasma and biometric changes promoted by fat-rich diet-induced obesity in this study, but restored intestinal morphometry. According to Sasaki et al.,^35^ sericin is a low digestibility protein remaining in the intestinal lumen for longer periods of time.^36^ Therefore, changes promoted by lipid accumulation may be reverted by the presence of sericin, which remains in contact with the intestinal mucosa. Sericin treatment also led to higher fecal excretion of lipids. Similar findings have been reported^16^ in a study with mice fed equivalent amounts of sericin and fibroin and a fat-rich diet. According to Limpeanchob et al.,^17^ sericin decreases cholesterol absorption in Caco-2 cells and micellar cholesterol solubilization, resulting in lower plasma cholesterol levels. Hence, sericin may interfere with absorption of dietary lipids in the small intestinal lumen, increasing lipid excretion in feces.

Other studies revealed that sericin may be protective against metabolic changes induced by obesogenic diets. The following effects have been reported: inhibition of weight gain and adipose mass deposit, improvement of plasma lipid profile and glucose tolerance, reduction of inflammatory markers (leptin, resistin and TNF-α), elevation of adiponectin levels, reduction of lipogenic enzyme activity in adipose and hepatic tissues and increase of beta-oxidation in the liver.^15 , 16^ In a recent study in rats with induced hypercholesterolemia and hyperglycemia, sericin decreased plasma cholesterol and reverted mitochondrial damage in the heart and liver. Attenuation of hepatic steatosis and hepatic oxidative stress were also observed, suggesting antioxidant properties.^18^

The effects of the protein sericin remain to be fully elucidated, particularly its therapeutic effects on fat-rich diet-induced obesity, a model that reflects the western lifestyle. Two of the changes promoted by the fat-rich diet in this study were reverted by treatment with sericin for 4 weeks; treatment for longer periods of time may have more significant effects. Also, given the small intestine is one of the first organs to adapt to obesogenic diets,^4^ reestablishment of intestinal morphometry suggests sericin may be a potential treatment for obesity.

## CONCLUSION

Intake of a fat-rich diet led to obesity development, as shown by biometric and plasmatic changes in treated mice, which were not reverted by treatment. The therapeutic dose of 1,000mg/kg of sericin derived from the *Bombyx mori* cocoon increased lipid excretion in feces and restored intestinal wall morphometry in obese mice.
