# Supplementary material for: Sericin as treatment of obesity: morphophysiological effects in obese mice fed with high-fat diet
Source: Einstein (Sao Paulo). 2019 Sep 20;18:eAO4876. doi: 10.31744/einstein_journal/2020AO4876 (PMC6905161; doi:10.31744/einstein_journal/2020AO4876)
Supplement: Translation

# Translation: Sericina como tratamento da obesidade: efeitos morfofisiológicos de camundongos obesos por dieta hiperlipídica

**Authors:** Regina Inês Kunz, Angélica Novi Capelassi, Ana Cláudia Paiva Alegre-Maller, Maria Lúcia Bonfleur, Lucinéia de Fátima Chasko Ribeiro, Rose Meire Costa, Maria Raquel Marçal Natali

**Affiliations:** 1 Brasil Universidade Federal da Fronteira Sul, Passo Fundo, RS, Brasil.; 2 Brasil Universidade Estadual do Oeste do Paraná, Cascavel, PR, Brasil.; 3 Brasil Universidade Estadual do Maringá, Maringá, PR, Brasil.

**Keywords:** Bombyx mori, Obesidade/tratamento farmacológico, Sericinas/uso terapêutico, Fígado, Intestino delgado, Jejuno, Dieta hiperlipídica, Camundongos

## Abstract

**Objetivo:**

Investigar os efeitos da sericina extraída de casulos de *Bombyx mori* na morfofisiologia de camundongos com obesidade induzida por dieta hiperlipídica.

**Métodos:**

Camundongos machos C57Bl6, com 9 semanas de idade, foram distribuídos em Grupos Controle e Obeso, que receberam ração padrão para roedores ou dieta hiperlipídica por 10 semanas, respectivamente. Posteriormente, os animais foram redistribuídos em quatro grupos, com sete animais cada: Controle, Controle-Sericina, Obeso e Obeso-Sericina. Os animais permaneceram recebendo ração padrão ou hiperlipídica por 4 semanas, período no qual a sericina foi administrada oralmente na dose de 1.000mg/kg de massa corporal aos Grupos Controle-Sericina e Obeso-Sericina. Parâmetros fisiológicos, como ganho de peso, consumo alimentar, peso das fezes em análise de lipídios fecais, motilidade intestinal e tolerância à glicose foram monitorados. Ao término do experimento, o plasma foi coletado para dosagens bioquímicas e fragmentos de tecido adiposo branco; fígado e jejuno foram processados para análises histológicas, e amostras hepáticas foram usadas para determinação lipídica.

**Resultados:**

Camundongos obesos apresentaram ganho de peso e acúmulo de gordura significativamente maior que os controles, aumento do colesterol total e glicemia. Houve hipertrofia dos adipócitos retroperitoneais e periepididimais, instalação de esteatose e aumento do colesterol e triglicerídeos hepáticos, bem como alteração morfométrica da parede jejunal.

**Conclusão:**

O tratamento com sericina não reverteu todas as alterações fisiológicas promovidas pela obesidade, mas restaurou a morfometria jejunal e aumentou a quantidade de lipídios eliminados nas fezes dos camundongos obesos, apresentando-se como potencial tratamento para a obesidade.

## INTRODUÇÃO

A obesidade, uma epidemia mundial, é caracterizada pelo excesso de gordura corporal acompanhada por complicações cardiometabólicas, que são diretamente impulsionadas pela ingesta excessiva de calorias, açúcar, sal e gordura, associada às atividades laborais e de entretenimento cada vez mais passivas.^1^

O intestino delgado, responsável pela absorção e pelo processamento metabólico eficiente dos nutrientes ingeridos,^2 , 3^ é o primeiro órgão exposto à energia derivada dos alimentos,^4^ capaz de se adaptar funcional e morfologicamente em relação à quantidade e à qualidade do alimento consumido.^5^ Sabe-se que a obesidade, principalmente a induzida por dietas hiperlipídicas e/ou hipercalóricas, afeta os componentes da parede intestinal.^3 , 5 , 6^

Somada às alterações no tecido adiposo e às eventuais adaptações no intestino delgado, a obesidade, por si só, é fator de risco para o desenvolvimento de outras patologias, como a doença hepática gordurosa não alcoólica (DHGNA) − a doença crônica mais comum do fígado.^7 , 8^

Mudanças no estilo de vida, como melhora nos hábitos alimentares e prática de atividade física, são as principais alternativas para o tratamento da obesidade e da DHGNA.^7 , 9^ Porém, a capacidade individual de resposta frente a estas estratégias pode não ser suficiente.^10^

Neste sentido, pesquisas de potenciais agentes terapêuticos naturais para a obesidade, com menos efeitos colaterais, vem ganhando destaque,^7 , 11^ como a do biopolímero sericina, produzido pelo bicho-da-seda *Bombyx mori* ( *Lepidoptera, Bombycidae* ). Trata-se de uma proteína globular natural, altamente hidrofílica, com peso molecular de 20 a 400kDa e que, em conjunto com a fibroína, forma o fio de seda.^12^ As características físico-químicas da molécula lhe conferem diversas propriedades, investigadas quanto à sua aplicabilidade biológica.^12 - 14^

Estudos evidenciaram que quando administrada concomitantemente à dieta hiperlipídica, a sericina melhora a tolerância à glicose e apresenta efeito hipolipidêmico,^15^ além de aumentar a concentração da lipoproteína de alta densidade (HDL) e regular a produção de citocinas pelo tecido adiposo, com redução da leptina, resistina e fator de necrose tumoral alfa (TNF-α), e aumento da concentração plasmática de adiponectina.^16^ Ainda, promove a inibição da absorção de colesterol pelas células intestinais^17^ e possui propriedade antioxidante.^18^ Neste sentido, é creditado à sericina amplo potencial como medida preventiva, mas não foi estabelecido seu efeito como terapia administrada após a instalação da obesidade e de suas comorbidades.

## OBJETIVO

Investigar os efeitos da sericina sobre os parâmetros plasmáticos e morfologia adiposa, hepática e intestinal de camundongos com obesidade induzida por dieta hiperlipídica.

## MÉTODOS

O estudo foi realizado na Universidade Estadual do Oeste do Paraná no período de julho de 2014 a dezembro de 2016.

### Extração e determinação aminoacídica da sericina

A sericina foi obtida a partir de casulos de *Bombyx mori* , cedidos pela indústria sericícola BRATAC S.A (Londrina, PR, Brasil). Os casulos foram cortados em pedaços de aproximadamente 1cm^2^ e submetidos ao processo de extração da sericina com o uso de alta temperatura e pressão em autoclave (CS 30, Prismatec, Itu, SP, Brasil), a 120°C e pressão de 1kgf/cm^2^ , por 60 minutos. A extração ocorreu sem adição de produtos químicos, utilizando-se 6g de casulo para cada 100mL de água destilada. A fibroína foi separada utilizando-se peneira de 18 malhas, e a solução de sericina foi congelada (-20°C) e, posteriormente, liofilizada (LT 1000, Terroni Equipamentos Ltda., São Carlos, SP, Brasil), obtendo-se a sericina em pó.

A determinação da composição de aminoácidos solúveis da sericina foi realizada por cromatografia líquida de ultraeficiência (UPLC), utilizando coluna analítica C18, fase reversa, acoplada a um detector ajustado no comprimento de onda de 214nm, no Laboratório de Bioquímica e Biofísica do Instituto Butantan (São Paulo, SP, Brasil).

### Animais e dieta

Todos os procedimentos utilizados foram previamente aprovados pelo Comitê de Ética Animal institucional (11 de abril de 2014).

Foram utilizados camundongos machos da linhagem C57Bl/6, com aproximadamente 9 semanas de vida e peso de 26,9±2,2g, mantidos em ambiente controlado (23 a 25°C e ciclo claro-escuro de 12 horas). Após 1 semana de aclimatação, foram divididos em dois grupos: Controle (n=14), que recebeu dieta padrão para roedores (Algomix^®^, Algomix Agroindustrial Ltda., Ouro Verde do Oeste, PR, Brasil) e água; e Obeso (n=14), que recebeu dieta hiperlipídica e água. Após 10 semanas de dieta, cada grupo foi subdividido, formando quatro grupos experimentais, com sete animais cada, submetidos ou não ao tratamento com sericina: Controle e Controle-Sericina; Obeso e Obeso-Sericina. Ao longo de todo o experimento, água e ração padrão ou hiperlipídica foram ofertados *ab libitum* .

Os ingredientes utilizados na composição da dieta hiperlipídica foram adquiridos na indústria Prag Soluções (Jaú, SP, Brasil). A ração foi preparada com a mistura dos componentes (30,07% de amido de milho, 14% de caseína, 12% de sacarose, 4% de óleo de soja, 5% de celulose microcristalina, 3,5% de mix mineral AIN-93M, 1,0% de mix vitamínico AIN-93M, 0,18% de L-cistina, 0,25% de bitartarato de colina e 30% de banha de porco). A ração foi ofertada aos animais na forma de pellets, contendo 32,21% de carboidratos, 10,6% de proteínas e 57,2% de lipídios.

O tratamento com sericina iniciou-se nos Grupos Controle-Sericina e Obeso-Sericina após 10 semanas das respectivas dietas e foi realizado diariamente, no mesmo horário, por 4 semanas consecutivas, utilizando-se a dose de 1.000mg/kg de peso corporal. A administração da sericina foi feita por gavagem, sendo a dose diária diluída em 300μL de salina 0,9%. Os animais dos Grupo Controle e Obeso foram gavados com 300μL de salina 0,9% pura.

### Pesagem dos animais, controle alimentar e coleta das fezes

A evolução do peso corporal foi obtida pela pesagem individual dos animais semanalmente. Na última semana de experimento, os animais foram alocados individualmente em gaiola metabólica por três períodos consecutivos de 12 horas, para a determinação do consumo alimentar. Posteriormente, calculou-se a média do consumo nos três períodos analisados. Após os três períodos de permanência na gaiola metabólica, as fezes dos animais foram coletadas, pesadas, congeladas e utilizadas para a determinação da quantidade total de lipídios.^19^

### Teste de trânsito intestinal

Quatro dias antes da eutanásia, 300µL de marcador (3g de carmin em 50mL de etilcelulose 0,5%) foram administrados por gavagem aos camundongos. O tempo decorrido da administração do marcador até a eliminação do primeiro pellet fecal colorido (vermelho-róseo) foi utilizado para determinação do trânsito intestinal.^20^

### Teste oral de tolerância à glicose

Dois dias antes da eutanásia e após 8 horas de jejum, os animais foram submetidos ao teste oral de tolerância à glicose (oGTT). Após a pesagem, foi realizada a coleta de sangue pela cauda, para obtenção da glicemia de jejum (tempo 0) com auxílio de glicosímetro (Accu-Chek Active^®^, Roche Diagnóstica Brasil Ltda., SP, Brasil). Após, os animais receberam glicose na concentração de 2g/kg de massa corporal por gavagem. A glicemia foi verificada aos 15, 30, 60 e 120 minutos após a administração da glicose. A área sob a curva (ASC) foi calculada para cada camundongo usando o software GraphPad Prism 6.0.

### Dosagens plasmáticas

Após jejum de 12 horas, os camundongos foram anestesiados intraperitonealmente com cloridrato de quetamina (100mg/kg) e xilazina (10mg/kg) e eutanasiados por exsanguinação do plexo orbital, com auxílio de capilar heparinizado. Após centrifugação (10.000rpm/10 minutos, a 4°C), o plasma foi retirado para dosagem de triglicerídeos e colesterol total, por meio de *kit* comercial, seguindo as instruções do fabricante (Laborclin^®^, Bioliquid, PR, Brasil), bem como determinação de alanina aminotransferase (ALT) e aspartato aminotransferase (AST), de acordo com as instruções do fabricante (Analisa^®^, Gold Analisa Diagnóstica Ltda., Belo Horizonte, MG, Brasil). A dosagem da glicemia foi obtida com auxílio do glicosímetro.

### Avaliação da obesidade

Ao final das 14 semanas experimentais, com aproximadamente 160 dias de vida, os animais foram pesados, e as gorduras retroperitoneal e periepididimal foram retiradas e pesadas, para determinação da instalação da obesidade.

### Estudos morfológicos

Amostras das gorduras retroperitoneal e periepididimal foram fixadas em paraformaldeído 4% em tampão fosfato-salina (PBS) por 24 horas, desidratadas em série crescente de álcool, diafanizadas em xilol e incluídas em parafina. Cortes de 5µm de espessura foram corados em hematoxilina e eosina (HE). Em seguida, com o auxílio do programa Image Pro Plus^®^6.0 (Media Cybernetics, USA), foi mensurada a área de 100 adipócitos por tipo de gordura por animal, distribuídos em 10 pontos aleatórios por corte.

O fígado foi excisado da cavidade abdominal e pesado para determinação da sua massa. Amostras do lobo direito foram coletadas e fixadas em paraformaldeído 4% em PBS por 24 horas, com posterior processamento para inclusão em parafina. Os cortes de 5µm de espessura foram corados em HE, para análise da morfologia geral, e tricrômico de Masson, para análise do tecido conjuntivo hepático. A determinação do grau de esteatose de zero a 3 foi adaptada de Kleiner et al.,^21^ sendo grau 0 se até 5%; grau 1 se 5 a 33%; grau 2 se 33 a 66%; e grau 3 se acima de 66% − sendo realizada avaliação duplo-cega.

O intestino delgado foi completamente estendido e mensurado quanto ao seu comprimento total. Amostras do jejuno foram abertas na borda mesentérica, fixadas em paraformaldeído 4% em PBS, e seguiram o procedimento histológico de rotina para inclusão em parafina. Os cortes semisseriados de 6µm de espessura foram corados com HE, para análise morfológica e morfométrica, e método histoquímico de ácido periódico-Schiff para evidenciação das células caliciformes.

As análises morfométricas do jejuno foram realizadas no programa Image Pro Plus^®^6.0 previamente calibrado. No aumento de 200x, foram mensuradas a espessura total da parede intestinal e a altura do vilo, em 10 pontos aleatórios por corte, separados entre si por 30µm, perfazendo 50 mensurações por animal. Já no aumento de 400x, foram mensuradas a espessura da túnica muscular, a profundidade da cripta e a largura do vilo, sendo realizadas 50 mensurações por animal para cada análise.

As células caliciformes foram quantificadas no vilo. Para tanto, o número de células caliciformes de um lado do vilo e o número total de células do mesmo lado do vilo foram contadas até um total de aproximadamente 1.500 células por animal. Assim, a porcentagem de células caliciformes foi calculada como o total de caliciformes corrigido pelo total de células contadas.

### Determinação de lipídios hepáticos

Fragmentos de aproximadamente 500mg de fígado por animal foram coletados e imediatamente congelados. Posteriormente, os lipídios hepáticos foram extraídos^19^ com determinação do colesterol e triglicerídeos, com os mesmos kits utilizados na determinação plasmática.

### Análise estatística

Os resultados foram expressos em média±erro padrão da média e analisados no programa GraphPad Prism 6.0. Após constatada a normalidade dos dados por meio do teste de Shapiro-Wilk, foi utilizado o teste de Análise de Variância (ANOVA) *two-way* com pós-teste de Tukey, adotando-se p<0,05 como estatisticamente significativo.

## RESULTADOS

### Composição de aminoácidos da sericina

A análise do perfil cromatográfico da sericina, realizada em UPLC, revelou a presença de 17 aminoácidos, com maior percentual de serina, glicina e ácido aspártico ( Tabela 1 ).

**Tabela 1:** Composição aminoacídica da sericina

Aminoácido	Percentual molar
Ácido aspártico	14,5
Ácido glutâmico	4,85
Serina	30,37
Glicina	27,16
Histidina	2,31
Arginina	2,55
Treonina	1,97
Alanina	0,73
Prolina	2,61
Tirosina	2,89
Valina	1,47
Metionina	0,19
Cisteína	0,84
Isoleucina	0,81
Leucina	2,54
Fenilalanina	0,17
Lisina	4,04

### Evolução do peso corporal

A partir da segunda semana de experimento, a ingestão de dieta hiperlipídica favoreceu o aumento estatisticamente significativo do peso corporal em relação aos animais que receberam ração padrão, resultado que se manteve ao longo das 14 semanas de experimento ( Figura 1A ). A oferta de ração hiperlipídica causou ingesta significativamente menor comparada à ração padrão ( Figura 1B ), embora tenha promovido o aumento da adiposidade, constatada pelas imagens da cavidade abdominal dos animais ( Figura 1C ).

**Figura 1:** Evolução do peso corporal. Gráfico da evolução do peso corporal (A) e consumo alimentar (B), e fotografias da cavidade abdominal aberta de camudongos C57Bl6 (C)

Resultados expressos por média±erro padrão da média (n=7/grupo). * p<0,01 em relação ao CT e CT-S. Análise de Variância *two-way* e pós-teste de Tukey. CT: controle; CT-S: controle-sericina; OB: obeso; OB-S: obeso-sericina.

### Teste de trânsito intestinal, peso das fezes e total de lipídios

A obesidade induzida por dieta e o tratamento com sericina não alteraram o tempo de trânsito intestinal dos animais ( Figura 2A ); entretanto, o volume total de fezes foi estatisticamente menor nos animais que receberam dieta hiperlipídica, o que esteve diretamente relacionado ao menor consumo alimentar observado nestes grupos, independente do tratamento com sericina ( Figura 2B ). Ainda, no Grupo Obeso-Sericina, houve aumento na quantidade total de lipídios fecais em comparação com as fezes dos animais alimentados com ração padrão ( Figura 2C ).

**Figura 2:** Análise do trânsito intestinal e parâmetros fecais. Teste de trânsito intestinal (A), peso das fezes (B), quantidade total de lipídios fecais (C)

Resultados expressos por média±erro padrão da média (n=7/grupo). * p<0,01 em relação ao CT e CT-S. Análise de Variância *two-way* e pós-teste de Tukey.

CT: controle; CT-S: controle-sericina; OB: obeso; OB-S: obeso-sericina.

### Teste oral de tolerância a glicose

Na figura 3A , verifica-se que no oGTT houve pico glicêmico aos 15 minutos para os animais de todos os grupos, com redução gradual da glicemia ao longo do teste e normalização aos 120 minutos. A ASC da glicemia não foi estatisticamente diferente nos grupos estudados ( Figura 3B ).

**Figura 3:** Resultado do teste oral de tolerância à glicose. Valores de glicemia durante o teste oral de tolerância à glicose (A), área sob da curva do teste oral de tolerância à glicose (B). Resultados expressos em média±erro padrão da média (n=7/grupo). Anova *Two Way* e pós-teste de Tukey

Resultados expressos por média±erro padrão da média (n=7/grupo). Análise de Variância *two-way* e pós-teste de Tukey.

CT: controle; CT-S: controle-sericina; OB: obeso; OB-S: obeso-sericina; GTT: teste oral de tolerância à glicose; ASC: área sob a curva.

### Validação do modelo de obesidade

Os parâmetros biométricos capazes de caracterizar a instalação da obesidade são apresentados na tabela 2 . O peso corporal final, o peso da carcaça e o acúmulo de gordura retroperitoneal e periepididimal foram influenciados somente pelo consumo de dieta hiperlipídica, indicando a instalação da obesidade, e o tratamento com sericina não influenciou nesses parâmetros.

**Tabela 2:** Parâmetros biométricos

Parâmetros biométricos	CT	CT-S	OB	OB-S
Peso corporal final, g	27,3±0,8	27,3±0,6	35,2±1,4*^†^	34,7±1,2*^†^
Peso da carcaça, g	19,9±0,7	20,3±0,6	25,6±1,2^*†^	25,1±1,0*^†^
Gordura retroperitoneal, % peso corporal	0,26±0,04	0,37±0,08	2,06±0,29*^†^	1,98±0,21*^†^
Gordura periepididimal, % peso corporal	0,72±0,06	0,82±0,09	3,92±0,29*^†^	3,66±0,50*^†^

Resultados expressos por média±erro padrão da média (n=7/grupo). Análise de Variância *two-way* e pós-teste de Tukey. * Diferença estatisticamente significativa em relação ao CT; p<0,01;^†^diferença estatisticamente significativa em relação ao CT-S; p<0,01.

CT: controle; CT-S: controle-sericina; OB: obeso; OB-S: obeso-sericina.

### Parâmetros plasmáticos

As concentrações plasmáticas de glicose e colesterol total foram influenciadas pelo consumo de dieta hiperlipídica, com aumento estatisticamente significativo nos animais obesos em relação aos controles, sendo que o tratamento com sericina não influenciou nesses parâmetros. Triglicerídeos e AST plasmáticas não diferiram entre os grupos, enquanto a concentração de ALT foi maior nos animais obesos quando comparada à dos animais que receberam ração padrão ( Tabela 3 ).

**Tabela 3:** Parâmetros plasmáticos

Parâmetros plasmáticos	CT	CT-S	OB	OB-S
Glicose, mg/dL	104,7±3,5	109,9±5,6	155,0±7,9*^†^	153,0±9,4*^†^
Colesterol, mg/dL	81,7±5,5	83,8±3,2	115,7±8,2*^†^	127,0±7,2*^†^
Triglicerídeos, mg/dL	55,4±6,7	58,1±3,0	55,6±4,0	50,7±2,4
ALT, U/L	13,9±2,7	15,0±1,2	29,1±3,8*^†^	30,2±6,3*^†^
AST, U/L	36,0±4,0	34,4±4,6	41,6±6,8	37,9±4,9

Resultados expressos por média±erro padrão da média (n=7/grupo). Análise de Variância *two-way* e pós-teste de Tukey. * Diferença estatisticamente significativa em relação ao CT; p<0,01;^†^diferença estatisticamente significativa em relação ao CT-S; p<0,01.

CT: controle; CT-S: controle-sericina; OB: obeso; OB-S: obeso-sericina; ALT: alanina aminotransferase; AST: aspartato aminotransferase.

### Morfologia do tecido adiposo

A área dos adipócitos retroperitoneais e periepididimais foi estatisticamente maior nos animais obesos, em relação aos alimentados com ração padrão ( Figuras 4A e 4B ). Apesar da discreta redução na área dos adipócitos no Grupo Obeso-Sericina, o tratamento com sericina não interferiu significativamente nesse parâmetro ( Figuras 4C e 4D ).

**Figura 4:** Morfometria e morfologia do tecido adiposo. Área dos adipócitos retroperitoneais (A) e periepididimais (B) fotomicrografias do tecido adiposo retroperitoneal (C) e periepididimal (D) de camundongos, coloração com hematoxilina e eosina

Resultados expressos por média±erro padrão da média (n=7/grupo). Análise de Variância *two-way* e pós-teste de Tukey. * p<0,01 em relação ao CT e CT-S.

CT: controle; CT-S: controle-sericina; OB: obeso; OB-S: obeso-sericina.

### Morfologia e análise de lipídios hepáticos

Não houve diferença estatística quanto ao peso do fígado entre os grupos ( Figura 5A ). A análise de lipídios hepáticos indicou maior quantidade de colesterol e triglicerídeos (p<0,0001) nos animais obesos em relação aos controles ( Figura 5B e 5C ). Nos camundongos dos Grupos Controle e Controle-Sericina, não foi verificada a presença de esteatose hepática, sendo os animais classificados como grau zero. Nos Grupos Obeso e Obeso-Sericina foi constatada a presença de esteatose, com variação dos graus obtidos na classificação ( Figura 5D ). Nos Grupos Obeso e Obeso-Sericina, além da presença de vesículas adiposas ( Figura 5E ), em alguns animais, o citoplasma dos hepatócitos se mostrou em aspecto de balão, com eventual formação de sincício entre células adjacentes.

**Figura 5:** Morfologia hepática e análise lipídica do fígado. Peso do fígado (A), análise do colesterol (B), e triglicerídeos (C) hepáticos; classificação de esteatose hepática (D), fotomicrografias do fígado de camundongos e coloração com hematoxilina e eosina (E)

Resultados expressos por média±erro padrão da média (n=7/grupo). Análise de Variância *two-way* e pós-teste de Tukey. * p<0,01 em relação ao CT e CT-S.

CT: controle; CT-S: controle-sericina; OB: obeso; OB-S: obeso-sericina.

### Morfometria intestinal

O consumo de dieta hiperlipídica alterou alguns parâmetros da morfometria intestinal, apresentados na tabela 4 . O comprimento do intestino delgado foi menor nos animais obesos em relação aos que receberam ração padrão. A altura do vilo foi estatisticamente maior no Grupo Obeso (p<0,01; p<0,05), enquanto houve redução da espessura da camada muscular (p<0,01; p<0,05) dos animais do Grupo Obeso em relação ao Controle e Controle-Sericina, respectivamente. Ainda, houve diminuição na profundidade da cripta nos animais obesos comparados aos controles (p<0,05). As alterações promovidas pelo consumo de dieta hiperlipídica foram revertidas pelo tratamento com sericina no Grupo Obeso-Sericina. A altura total da parede intestinal, largura do vilo e índice de células caliciformes não diferiu entre os grupos estudados.

**Tabela 4:** Morfometria intestinal

Morfometria intestinal	CT	CT-S	OB	OB-S
Comprimento do intestino delgado, cm	39,7±0,9	39,9±0,6	33,1±0,9*^†^	33,8±0,9*^†^
Espessura total da parede intestinal, µm	496,4±17,9	504,7±24,0	558,7±30,6	513,0±17,7
Altura do vilo, µm	306,0±19,9	327,9±13,6	390,2±18,8*^†^	356,4±8,4
Profundidade da cripta, µm	96,2±3,0	90,2±4,0	78,5±5,6*	84,2±4,7
Largura do vilo, µm	94,0±2,3	95,5±2,5	91,0±1,2	90,5±1,6
Espessura da túnica muscular, µm	51,1±3,6	50,1±3,87	37,2±1,6*^†^	41,1±1,9
Células caliciformes, %	5,0±0,3	4,7±0,3	4,4±0,3	4,2±0,3

Resultados expressos por média±erro padrão da média (n=7/grupo). Análise de Variância *two-way* e pós-teste de Tukey. * Diferença estatisticamente significativa em relação ao CT;^†^diferença estatisticamente significativa em relação ao CT-S.

CT: controle; CT-S: controle-sericina; OB: obeso; OB-S: obeso-sericina.

## DISCUSSÃO

O camundongo C57Bl/6 é um modelo animal suscetível ao desenvolvimento de obesidade induzida por dieta.^22^ Constatamos que, apesar da menor ingesta, a dieta hiperlipídica foi eficaz na indução da obesidade, promovendo aumento do peso corporal, acúmulo de gordura e maior concentração plasmática de colesterol e glicose nos camundongos. Esses dados estão de acordo com a literatura atual,^23 - 26^ segundo a qual o consumo de dietas ricas em lipídios saturados, como a usada neste estudo, induzem à obesidade.

Apesar do maior consumo de lipídios, não houve aumento na concentração plasmática de triglicerídeos nos animais obesos, resultado encontrado também em camundongos alimentados com dieta hiperlipídica^24 - 26^ e em ratos.^23^ Provavelmente, a concentração sérica de triglicerídeos não foi alterada devido ao redirecionamento dessas moléculas para outros tecidos, principalmente o tecido adiposo, no qual são armazenados.^27^ Hipertrofia ou hiperplasia costumam ser respostas dos adipócitos frente ao acúmulo de triglicerídeos.^28^ Constatamos o aumento da massa de gordura depositada nas regiões retroperitoneal e periepididimal, bem como aumento na área dos adipócitos dos animais obesos. Além da hipertrofia, os adipócitos podem sofrer com alterações na homeostase celular, como hipóxia, infiltração de macrófagos, fibrose e resistência à insulina no tecido adiposo, condições que podem culminar na síndrome metabólica.^29^

Partículas remanescentes de gordura são removidas da circulação pelo fígado,^27^ que passa a acumular quantidades significativas de lipídios em indivíduos obesos, caracterizando a DHGNA.^8^ Verificamos que houve aumento da quantidade de triglicerídeos e colesterol hepáticos, bem como o acúmulo de gordura na forma de vesículas no fígado dos camundongos obesos. O aparecimento de vesículas brancas na coloração em HE do fígado comprova a esteatose hepática,^25^ condição morfológica também evidenciada em camundongos^24^ e em ratos.^18^ A análise bioquímica revelou aumento na concentração sérica de ALT nos camundongos obesos, embora estudos recentes difiram quanto ao efeito da obesidade sobre as transaminases hepáticas, visto que os mesmos não são unânimes quanto ao uso dos modelos animais e às dietas utilizadas. Sung et al.,^25^ não verificaram alterações nas concentrações das transaminases, enquanto Choi et al.,^30^ não observaram mudanças na concentração de ALT. Pang et al.,^26^ só observaram aumento de AST nos camundongos obesos por dieta hiperlipídica. Ainda, Abd El-Kader et al.,^8^ ressaltam que as enzimas hepáticas podem estar normais ou minimamente alteradas na DHGNA.

Além de seu papel essencial na digestão e na absorção dos nutrientes, o intestino delgado pode rapidamente alterar seu metabolismo em resposta ao maior conteúdo luminal de lipídios,^4^ o que pode estar relacionado com a progressão da síndrome metabólica em outros órgãos.^31 , 32^ A oferta de dieta hiperlipídica promoveu a redução no comprimento do intestino delgado e o aumento na altura dos vilos, associados à diminuição da profundidade da cripta e da espessura da túnica muscular no jejuno dos animais estudados. Com exceção da espessura da túnica muscular, resultados morfométricos similares foram previamente encontrados em camundongos *Swiss* alimentados com dieta hiperlipídica por 8 semanas.^6^ Como o consumo alimentar dos animais obesos foi menor, as alterações na mucosa não podem ser relacionadas à hiperfagia,^5^ e sim ao tipo de alimento.^3^ A maior área de absorção intestinal promovida pelo aumento da altura do vilo^5^ provavelmente resultou no ganho de peso dos animais obesos. Dietas de consistência mais líquida, como a hiperlipídica, podem não alterar a morfologia da cripta, ao mesmo tempo em que promovem redução da espessura da túnica muscular,^33^ como verificado no presente estudo, devido à provável diminuição da motilidade do trato gastrintestinal. Evidências experimentais têm demonstrado que dietas hiperlipídicas podem ainda causar perda neuronal no plexo mioentérico,^34^ promovendo redução da espessura muscular, e, consequentemente, reduzir a motilidade intestinal. Apesar da aparente redução do peristaltismo necessário à digestão e à absorção da dieta hiperlipídica, o tempo de trânsito intestinal e o índice de células caliciformes, produtoras de muco para a proteção e lubrificação do epitélio intestinal,^3^ não foram diferentes entre os grupos estudados, demonstrando que não houve alteração na função.

A sericina utilizada no presente estudo foi extraída sem utilização de qualquer aditivo químico, obtendo-se um produto final sem impureza^18^ e composto por 17 aminoácidos, sendo que serina, glicina e ácido aspártico correspondem a mais de 72% da proteína. Composição aminoacídica semelhante foi descrita anteriormente,^13 , 15 , 17^ confirmando a característica hidrofílica da sericina. Quando administrada aos camundongos do Grupo Controle-Sericina, a proteína não promoveu alteração plasmática ou morfológica nos tecidos analisados, confirmando o uso seguro para consumo.^16 , 35^

Apesar de não ter revertido as alterações plasmáticas e biométricas promovidas pela obesidade induzida por dieta hiperlipídica, o tratamento com sericina restaurou a morfometria intestinal. Segundo Sasaki et al.,^35^ a sericina é uma proteína de baixa digestibilidade, que pode permanecer por maior tempo no lúmen intestinal.^36^ Assim, as alterações promovidas pelo acúmulo de lipídios são revertidas pela presença de sericina, que está em contato com a mucosa intestinal. Além disso, verificamos que o tratamento com sericina promoveu maior excreção de lipídios nas fezes. Resultado similar foi encontrado previamente,^16^ utilizando quantidades equivalentes de sericina e fibroína em camundongos alimentados com dieta hiperlipídica. Segundo Limpeanchob et al.,^17^ a sericina reduz a absorção de colesterol em células Caco-2, bem como sua solubilização em micelas, promovendo a diminuição da concentração plasmática de colesterol. Assim, a presença de sericina no lúmen do intestino delgado parece interferir na absorção dos lipídios da dieta, que são, então, eliminados em maior quantidade nas fezes.

Outros estudos verificaram que o consumo de sericina pode exercer efeito protetor às alterações metabólicas promovidas por dietas obesogênicas. Dentre os efeitos, podem ser destacados: inibição do ganho de peso e acúmulo de massa adiposa, melhora do perfil lipídico plasmático e da tolerância à glicose, redução da concentração de mediadores inflamatórios (leptina, resistina e TNF-α) e aumento de adiponectina, bem como diminuição da atividade de enzimas lipogênicas no tecido adiposo e hepático, associado ao aumento da betaoxidação no fígado.^15 , 16^ Recentemente, em um estudo com ratos com hipercolesterolemia e hiperglicemia induzidas, a sericina reduziu o colesterol plasmático e reverteu os danos mitocondriais no coração e fígado. Ainda, atenuou a esteatose hepática, ao mesmo tempo em que diminuiu o estresse oxidativo no órgão, características que podem ser associadas à propriedade antioxidante da sericina.^18^

Cabe ressaltar que ainda não estão completamente elucidados na literatura os efeitos da proteína sericina, sobretudo quando aplicada como medida terapêutica na obesidade induzida por dieta hiperlipídica, modelo que reproduz o estilo ocidental. Duas alterações promovidas pela dieta hiperlipídica no presente estudo foram revertidas pelo uso de sericina por 4 semanas, de modo que maior tempo de tratamento pode revelar resultados mais significativos. Ainda, considerando que o intestino delgado é um dos primeiros órgãos a se adaptar às dietas obesogênicas,^4^ o reestabelecimento da morfometria intestinal indica que a sericina apresenta potencial tratamento para a obesidade.

## CONCLUSÃO

O consumo de dieta hiperlipídica foi responsável pela instalação da obesidade, caracterizada por alterações biométricas e plasmáticas nos camundongos, que não foram revertidas pelo tratamento. A dose de 1.000mg/kg de sericina, extraída do casulo de *Bombyx mori* , promoveu maior eliminação de lipídios fecais, bem como restabeleceu a morfometria da parede jejunal nos camundongos obesos.
